# Clinical outcomes of frozen–thawed blastocysts from zygotes with no or one pronucleus for in vitro fertilization and intracytoplasmic sperm injection cycles

**DOI:** 10.1007/s00404-023-07118-1

**Published:** 2023-06-30

**Authors:** Xiaomei Tong, Jiamin Jin, Yamei Xue, Lu Fang, Haiyan Zhu, Lingying Jiang, Songying Zhang

**Affiliations:** 1 Assisted Reproduction Unit, Department of Obstetrics and Gynecology, Sir Run Run Shaw Hospital, Zhejiang University School of Medicine, Shangcheng District, No. 3 Qingchun East Road, Hangzhou, 310016 China; 2 Key Laboratory of Reproductive Dysfunction Management of Zhejiang Province, Hangzhou, 310016 China

**Keywords:** IVF, ICSI, Blastocyst transfer, Live birth rate, PGT-A

## Abstract

**Purpose:**

To investigate the clinical outcomes of in vitro fertilization (IVF) and intracytoplasmic sperm injection (ICSI) cycles using frozen–thawed blastocyst transfers derived from zygotes with no (0PN) or one pronucleus (1PN).

**Methods:**

This retrospective study included 7084 0PN, 2238 1PN, and 72,266 two pronuclear (2PN) embryos cultured to the blastocyst stage from 19,631 IVF and 12,377 ICSI cycles between March 2018 and December 2021. Developmental potential and clinical outcomes of 0PN, 1PN, and 2PN embryos were analyzed. A total of 290 0PN-, 92 1PN-, and 1906 2PN-derived single frozen–thawed blastocyst transfers were performed. Chromosome euploid rates of 0PN-, 1PN-, and 2PN-derived blastocysts were analyzed by next-generation sequencing. Euploid 0PN- and 1PN-derived blastocysts underwent subsequent Infinium Asian Screening Array gene chip analysis to detect ploidy alterations.

**Results:**

Available blastocyst rates of 0PN and 1PN embryos were significantly lower than those of 2PN embryos in both IVF and ICSI cycles. Single 0PN and 1PN blastocysts transferred in frozen–thawed cycles resulted in a similar clinical pregnancy rate, miscarriage rate, live birth rate, and neonatal outcome to 2PN blastocysts in IVF and ICSI cycles. Genetic analysis showed that euploid rates of 0PN- and 1PN-derived blastocysts used for ICSI cycles were similar to that of 2PN-derived blastocysts.

**Conclusion:**

Our study indicated that 0PN- and 1PN-derived blastocysts resulted in similar clinical outcomes to 2PN-derived blastocysts. The 0PN- and 1PN-derived blastocysts from ICSI cycles can be transferred as well as those from IVF cycles when the number of 2PN-derived blastocysts is insufficient.

## What does this study add to the clinical work

**Table:** 

The 0PN and 1PN embryos reaching blastocyst stage from ICSI cycles could achieve similar clinical outcomes of frozen-thawed embryo transfers as blastocysts from IVF. This study further strengthens the potential use of 0PN and 1PN blastocysts from ICSI cycles.	

## Introduction

Assessment of in vitro fertilization (IVF) is generally performed at 16–18 h after the fertilization procedure [1]. Typically, fertilized oocytes exhibiting two pronuclei (2PN) under light microscopy are cultured further and selected for transfer. Mature oocytes without a pronucleus (0PN) or with one pronucleus (1PN) are generally considered to be a failed or abnormal fertilization. It has been reported that 11.3%–20.0% and 1.6%–7.7% of 0PN and 1PN zygotes, respectively, are present after IVF and intracytoplasmic sperm injection (ICSI) [2, 3]. Although embryos that develop from 0PN and 1PN zygotes are capable of developing into blastocysts in vitro and may result in healthy babies, they are usually discarded because of a possible higher proportion of chromosomal abnormalities [4–7]. Some studies of the genetic characteristics of 0PN and 1PN embryos have suggested that a considerable number of 0PN and 1PN embryos have a normal chromosomal composition, and thus may be considered for transfer when no embryos develop from 2PN zygotes [8–10]. 0PN and 1PN cleavage stage embryos resulted in a lower implantation rate (IR) and live birth rate (LBR) compared with 2PN cleavage stage embryos [11, 12], whereas IR and LBR were comparable between 0PN/1PN- and 2PN-derived blastocyst transfers [12, 13]. However, some studies suggest that 0PN- and 1PN-derived blastocyst transfers resulted in a lower IR and LBR than 2PN-derived blastocysts, especially in ICSI cycles [14, 15]. Thus, the clinical outcomes of 0PN- and 1PN-derived blastocysts from different fertilization methods need to be investigated further.

In this study, we conducted a retrospective analysis of blastocyst formation of 0PN and 1PN embryos from IVF and ICSI cycles. The clinical outcomes of single 0PN- and 1PN-derived frozen–thawed blastocyst transfers were compared. The genetic results of 0PN- and 1PN-derived blastocysts of preimplantation genetic testing for aneuploidy (PGT-A) cycles were also analyzed. The aim of this study was to investigate whether 0PN- and 1PN-derived blastocysts from ICSI cycles are comparable with blastocyst transfers from IVF cycles.

## Methods

### Study design

This study was performed at the Department of Reproductive Medicine, Sir Run Run Shaw Hospital affiliated with Zhejiang University School of Medicine. The available blastocyst rates of 7084 0PN, 2238 1PN, and 72,266 2PN embryos from 19,631 IVF and 12,377 ICSI cycles cultured to the blastocyst stage from March 2018 to December 2021 were analyzed. The clinical outcomes after transfer of 0PN-, 1PN-, and 2PN zygote-derived single frozen–thawed blastocysts (hereafter denoted as 0PN, 1PN, and 2PN blastocysts, respectively) were compared. The chromosomal characteristics of 0PN, 1PN, and 2PN blastocysts from PGT-A cycles were analyzed. All PGT-A cycles underwent ICSI treatment. This study was approved by the medical ethical committee of Sir Run Run Shaw Hospital.

### Evaluation of fertilization, and embryo culture and handling

Patients underwent controlled ovarian stimulation as described previously [16]. Sequential culture media (Cook Medical, Sydney, Australia) were used for embryo culture. 0PN, 1PN, and 2PN zygotes were cultured further. Embryos on day 3 was evaluated for blastomere number and embryo morphology in accordance with Peter’s scoring system [17]. Embryos with more than four blastomeres and cytoplasmic fragmentation less than 50% were cultured to the day 5 and 6 blastocyst stage. Formed blastocysts graded as 3–6 with an inner cell mass (ICM) greater than grade B or with ICM grade C and trophoblast cell (TE) greater than grade B in accordance with Gardner’s grading system [18] were selected for vitrification. Blastocysts graded as 4–6 with ICM and TE scores both equal to or better than grade B were considered to be high-quality blastocysts. Single blastocyst transfer was performed after warming the vitrified blastocysts. In PGT-A cycles, TE biopsy was performed on day 5 and 6 blastocysts without prehatching, so that embryos were undisturbed until the expanded blastocyst stage. Three–five TE cells from the opposite side of the ICM were dissected and separated from blastocysts by laser combined with mechanical dissection.

### Genetic analysis of blastocysts

Whole-genome amplification (WGA) of biopsied TE cell samples was performed using a Sample Processing Kit for Gene Sequencing (Yikon Genomics, Suzhou, China) in accordance with the manufacturer’ s instructions. WGA products were used for library construction with a library size of 200–500 bp, generating approximately 2 million raw reads for each TE sample [19]. The WGA products were then sequenced using an Illumina Nextseq 550 platform (Illumina, San Diego, CA, USA). Whole-genome copy number variations were analyzed to determine the euploidy or aneuploidy status of TE cell samples. WGA products from euploid 0PN and 1PN blastocysts and the genome of parental peripheral blood cells were analyzed using Illumina Infinium Asian Screening Array-24 + v1.0 (ASA) gene chips. Single-nucleotide polymorphisms of TE cell samples and parent cells were analyzed to exclude ploidy abnormalities such as polyploid, haploid, and uniparental disomy.

### Follow-up

Serum human chorionic gonadotropin β-subunit levels were evaluated at 10 days after blastocyst transfer. The presence of a gestation sac by ultrasound after 5 weeks was defined as clinical pregnancy. Live birth was defined as at least one live baby born after 28 weeks of gestation, and the implantation rate was calculated as the number of gestational sacs per number of embryos transferred. Miscarriage was defined as loss of a previously visualized intrauterine gestational sac by ultrasound before 28 weeks. Gestational age was calculated by adding 20 days from the date of blastocyst transfer. Preterm delivery was defined as delivery at < 37 weeks of gestation. Congenital malformations at birth were diagnosed in accordance with the Chinese Birth Defects Monitoring Program. Data on pregnancy loss and delivery were acquired through telephone interviews.

### Statistical analysis

Statistical analyses were performed using SPSS software version 19.0. For mean values, the independent sample t-test and variance analysis were used to assess inter- and intra-group differences, respectively. For proportional values, the chi-squared test and chi-squared test of multiple comparisons were used to assess inter- and intra-group differences, respectively. A *P* value of < 0.05 was considered as statistically significant.

## Results

### The blastocyst formation from 0PN and 1PN embryos in IVF and ICSI cycles

In both IVF and ICSI cycles, the available blastocyst rate of 0PN embryos was lower than that of 2PN embryos (47.7% vs. 53.4% in IVF, *P* < 0.05; 32.0% vs. 49.8% in ICSI, *P* < 0.05; Table 1). However, the rates of day 5 blastocysts and high-quality blastocysts from 0PN embryos were higher than those from 2PN embryos (85.5% vs. 69.6% and 56.1% vs. 44.7%, respectively, in IVF, *P* < 0.05; 75.5% vs. 61.6% and 57.1% vs. 42.9%, respectively, in ICSI, *P* < 0.05).

**Table 1:** Available blastocyst formation from embryos with no (0PN), one (1PN) and two pronucleus (2PN) for IVF and ICSI cycles

	IVF			*P* value	ICSI			*P* value	*P* value (IVF vs. ICSI)		
	0PN	1PN	2PN		0PN	1PN	2PN		0PN	1PN	2PN
No. of embryos cultured to blastocyst stage (*n*)	5708	1767	42,955	N/A	1376	471	29,311	N/A	N/A	N/A	N/A
Available blastocyst rate (*n*, %)	2725 (47.7%)^a^	510 (28.9%)^b^	22,952 (53.4%)^c^	0.000	441 (32.0%)^a^	76 (16.1%)^b^	14,594 (49.8%)^c^	0.000	0.000	0.000	0.000
Day 5 blastocysts (*n*, %)	2331 (85.5%)^a^	359 (70.4%)^b^	15,980 (69.6%)^b^	0.000	333 (75.5%)^a^	36 (47.4%)^b^	8987 (61.6%)^c^	0.000	0.000	0.000	0.000
High-quality blastocysts (*n*, %) (D5 + D6)	1528 (56.1%)^a^	254 (49.8%)^b^	10,250 (44.7%)^b^	0.000	252 (57.1%)^a^	29 (38.2%)^b^	6264 (42.9%)^b^	0.000	0.674	0.058	0.001

Data are presented as *n* (%)

For inter-group comparison of IVF and ICSI cycles, different superscripts in the same row indicate significant differences (*P* < 0.05), and the same superscripts indicate no significant difference (*P* > 0.05). N/A, not applicable; D5 + D6, day 5 and day 6

The available blastocyst rate of 1PN embryos was also decreased in both IVF and ICSI cycles, when compared with 2PN embryos (28.9% vs. 53.4% in IVF, *P* < 0.05; 16.1% vs. 49.8% in ICSI, *P* < 0.05, respectively). The rate of day 5 blastocysts from 1PN embryos was lower than those from 2PN embryos in ICSI cycles (47.4% vs 61.6%, *P* < 0.05). The rates of day 5 blastocysts in IVF cycles and high-quality blastocysts in both IVF and ICSI cycles did not differ between 1PN and 2PN embryos.

The rates of available blastocysts and day 5 blastocysts from 0PN and 1PN embryos were much lower in ICSI cycles than IVF cycles (*P* < 0.05). The fertilization methods significantly influenced blastocyst formation from 0PN and 1PN embryos.

### Clinical outcomes of transfer single frozen–thawed 0PN and 1PN blastocysts

In this study, 290 0PN and 92 1PN single frozen–thawed blastocyst transfers were performed, resulting in 106 0PN- and 35 1PN-derived singletons, respectively, compared with 1906 2PN single blastocyst transfers and 815 2PN-derived singletons, respectively. We compared the clinical outcomes of 0PN and 1PN blastocyst transfers with 2PN blastocyst transfers in IVF and ICSI cycles. As shown in Table 2, the clinical pregnancy rates (CPR) of 0PN, 1PN and 2PN blastocyst transfers in IVF cycles were 46.2%, 52.6% and 50.7%, respectively; the LBRs were 36.1%, 37.2% and 42.3%, respectively; and the miscarriage rates (MRs) were 20.0%, 26.8% and 16.2%, respectively. The CPR, LBR and MR did not differ significantly among the three groups. Furthermore, no significant differences in the gestational age, birthweight or congenital malformations at birth were observed in 0PN and 1PN blastocyst transfers when compared with 2PN blastocyst transfers. The major pregnancy complications and mode of delivery were also similar among the three groups, as shown in supplementary table 1.

**Table 2:** Clinical outcomes of no (0PN), one (1PN) and two pronucleus (2PN) embryo-derived single frozen–thawed blastocyst transfers in IVF and ICSI cycles

	IVF			*P* value	ICSI			*P* value	*P* value (IVF vs. ICSI)		
	0PN	1PN	2PN		0PN	1PN	2PN		0PN	1PN	2PN
No. of transferred cycles	249	78	1366	N/A	41	14	540	N/A	N/A	N/A	N/A
Endometrial thickness (mm)	9.41 ± 1.57^a^	9.31 ± 1.78^a^	9.32 ± 1.51^a^	0.700	9.65 ± 1.75^a^	9.38 ± 2.54^a^	9.30 ± 1.56^a^	0.415	0.384	0.902	0.792
Clinical pregnancy (*n*, %)	115 (46.2%)^a^	41 (52.6%)^a^	692 (50.7%)^a^	0.389	19 (46.3%)^a^	6 (42.9%)^a^	266 (49.3%)^a^	0.844	0.985	0.503	0.582
Miscarriage (*n*, %)	23 (20.0%)^a^	11 (26.8%)^a^	112 (16.2%)^a^	0.150	3 (15.8%)^a^	0 (0)^a^	27 (10.2%)^a^	0.518	0.667	0.147	0.018
Live birth (*n*, %)	90 (36.1%)^a^	29 (37.2%)^a^	578 (42.3%)^a^	0.146	16 (39.0%)^a^	6 (42.9%)^a^	237 (43.9%)^a^	0.831	0.723	0.687	0.531
Mean gestational age (weeks)	38.12 ± 1.51^a^	38.00 ± 1.44^a^	38.01 ± 1.95^a^	0.733	38.06 ± 2.46^a^	37.17 ± 5.60^a^	38.22 ± 1.42^a^	0.138	0.896	0.472	0.140
Preterm delivery (< 37 weeks) (*n*, %)	8 (8.9%)^a^	3 (10.3%)^a^	83 (14.4%)^a^	0.324	3 (18.8%)^a^	1 (16.7%)^a^	23 (9.7%)^a^	0.456	0.233	0.658	0.073
Term delivery (≥ 37 weeks) (*n*, %)	82 (91.1%)^a^	26 (89.7%)^a^	495 (85.6%)^a^		13 (81.2%)^a^	5 (83.3%)^a^	214 (90.3%)^a^				
Birthweight (g)	3236.33 ± 512.13^a^	3200.69 ± 492.14^a^	3316.25 ± 544.84^a^	0.258	3283.13 ± 746.55^a^	3285.83 ± 1177.24^a^	3322.52 ± 480.02^a^	0.859	0.756	0.770	0.877
Congenital malformations at birth (*n*, %)	0 (0.0%)^a^	0 (0.0%)^a^	1 (0.2%)^a^	0.902	0 (0.0%)^a^	0 (0.0%)^a^	2 (0.8%)^a^	0.911	N/A	N/A	0.151

Data are presented as mean ± standard deviation or *n* (%)

*N/A* not applicable

Forty-one 0PN and 14 1PN blastocysts were transferred in ICSI cycles (Table 2). The 0PN blastocyst transfers resulted in a CPR of 46.3%, a LBR of 39.0% and a MR of 15.8%. The 1PN blastocyst transfers resulted in a CPR of 42.9% and all clinical pregnancies achieved live birth. No significant differences in CPR, LBR or MR for 0PN and 1PN blastocyst transfers were observed when compared with 2PN blastocyst transfers. The gestational age, birthweight, or congenital malformations of newborns did not differ significantly among the three groups.

### Genetic analysis of 0PN and 1PN blastocysts derived from ICSI cycles

To investigate whether there was a decrease in euploid rates of 0PN and 1PN blastocysts derived from ICSI cycles, we evaluated TE biopsy samples from 64 0PN, 20 1PN, and 2009 2PN blastocysts that underwent PGT-A. ICSI was performed in all PGT-A cycles. The WGA rate was similar among the three groups (Table 3). The euploid rates of 0PN, 1PN, and 2PN blastocysts were 62.5%, 60.0%, and 54.4%, respectively. Euploid, aneuploid, and mosaic rates of 0PN and 1PN blastocysts were comparable with those of 2PN blastocysts. No ploidy abnormalities were found in euploid 0PN or 1PN blastocysts by ASA gene chip analysis.

**Table 3:** The chromosomal aneuploidies of no pronucleus (0PN), one pronucleus (1PN) and two pronuclear (2PN) embryo-derived blastocysts from ICSI cycles

	0PN	1PN	2PN	*P* value
No. of blastocysts for PGT-A	64	20	2009	N/A
Whole-genome amplification rate (%)	100.0%	100.0%	99.2%	0.714
Euploid blastocysts (*n*, %)	40 (62.5%)	12 (60.0%)	1093 (54.4%)	0.393
Aneuploid blastocysts (*n*, %)	23 (35.9%)	8 (40.0%)	749 (37.3%)	0.945
Mosaic blastocysts (*n*, %)	1 (1.6%)	0 (0.0%)	151 (7.5%)	0.089

Data are presented as *n* (%)

*N/A* not applicable

## Discussion

In this study, 0PN and 1PN embryos exhibited a lower available blastocyst rate than 2PN embryos, especially in ICSI cycles. Single 0PN and 1PN blastocysts transferred in frozen–thawed cycles resulted in a similar CPR, LBR, and neonatal outcome compared with 2PN blastocysts in both IVF and ICSI cycles. No significant differences were observed in chromosomal euploid rates for 0PN, 1PN, or 2PN blastocysts in ICSI cycles.

The availability of 0PN and 1PN embryos for clinical use is currently disputed. They are generally selected for use when only 2PN embryos are unavailable. However, the clinical outcomes of 0PN and 1PN embryo transfers vary significantly among IVF clinics, and the influence of different fertilization methods remains unclear.

0PN may result from the lack of formation of PN, disappearance of PN, or unfertilized oocytes [15]. Previous studies have reported that the cleavage rate of 0PN zygotes is lower than that of 2PN zygotes. The use of time-lapse monitoring allows for sequential and accurate observation of PN and facilitates determining the origin of 0PN zygotes [20]. A recent study performed time-lapse monitoring for PN assessment and revealed that the PN in 7.59% of zygotes had already disappeared at 20 h after fertilization, indicating that PN may not be observed in these fertilized zygotes at a fixed time point and misidentified as 0PN zygotes in an ordinary incubator [20]. This may explain why some 0PN zygotes have a normal 2PN origin and the potential to develop into blastocysts and implant as 2PN zygotes.

Chen et al. reported a decrease in the available blastocyst rates of 0PN embryos compared with those of 2PN embryos, and the cell number of 0PN embryos on day 3 affected subsequent blastocyst formation [21]. The available blastocyst rate of 0PN embryos was higher for ≥ 6-cell than < 6-cell day 3 embryos. The current results demonstrated that 47.7% and 32.0% of 0PN embryos in IVF and ICSI cycles, respectively, developed to the blastocyst stage. This development was significantly lower than that of 2PN embryos, and the rate was lower in ICSI cycles compared with IVF cycles. In addition to normal fertilization, cleaved 0PN embryos may also be due to abnormal fertilization (1PN and > 2PN) or parthenogenetic activation and cannot develop to the blastocyst stage [12]. We speculate that 0PN embryos from ICSI cycles may have a higher rate of abnormal fertilization or parthenogenetic activation than those from IVF cycles. 0PN embryos from ICSI cycles may originate from other mechanisms associated with male factors or microinjection rather than early PN disappearance. It has been reported that extending the culture to blastocyst stage may reduce the number of embryos with abnormal chromosomes, when embryos complete transformation from maternal to embryo-derived genes [22–24]. In the present study, the CPR and LBR of single frozen–thawed 0PN blastocysts obtained from IVF and ICSI cycles were nearly the same as those of 2PN blastocysts. No significant differences were found in the gestational age, birthweight, or congenital malformations of newborns. These results suggest that 0PN blastocysts derived from both IVF and ICSI cycles can be transferred and produce equivalent pregnancy outcomes to 2PN blastocysts.

We used NGS to assess chromosomal abnormalities of 0PN, 1PN, and 2PN blastocysts that underwent PGT-A. However, NGS was unable to detect ploidy alterations. As a result, haploid or polyploid cells may be present in blastocysts diagnosed as euploid by NGS. The ASA gene chip was used to detect the ploidy abnormalities of 0PN and 1PN blastocysts. There were no significant differences in the euploid rates of 0PN and 2PN blastocysts from ICSI cycles, and no ploidy abnormalities were observed in euploid 0PN blastocysts. Our findings were consistent with previous results showing no significant differences in chromosomal abnormalities between 0PN and 2PN blastocysts [25, 26].

1PN may result from inappropriate timing of PN formation and/or disappearance of PN, fusion of paternal and maternal PN, or parthenogenetic activation [27, 28]. Time-lapse monitoring can be used to help rule out the appearance of 1PN due to asynchronous PN formation or PN fusion. In previous studies, 1PN embryos had a reduced developmental potential compared with 2PN embryos [29, 30]. The blastocyst formation rates of 1PN and 2PN embryos were 14.8% and 36.4%, respectively, in IVF cycles, and 6.6% and 34.0%, respectively, in ICSI cycles [30]. The results from the present study were similar. Specifically, 28.9% of 1PN embryos from IVF cycles developed into available blastocysts, whereas only 16.1% of 1PN embryos from ICSI cycles developed into available blastocysts. This may be related to the different mechanisms of 1PN formation between IVF and ICSI cycles. Some studies have reported that the diploid rate of 1PN zygotes from IVF cycles is higher than that of 1PN zygotes from ICSI cycles [8, 10]. 1PN zygotes from IVF cycles are mainly formed by exclosure of juxtaposed male and female pronuclei in a common pronuclear envelope [28]. However, the rate of haploid 1PN zygotes in ICSI cycles is higher than that of haploid 1PN zygotes in IVF cycles [10, 31]. Palermo et al. found that only 10% of 1PN zygotes from the ICSI cycles contained Y chromosome, suggesting that the 1PN zygotes were usually derived from parthenogenesis [32]. These parthenogenetically activated embryos might be able to develop to the cleavage stage, but rarely to the blastocyst stage. Blastocyst culture would be helpful to select diploid 1PN embryos for transfer.

Bradley et al. analyzed 74 IVF and 32 ICSI 1PN blastocysts for PGT-A by comparative genomic hybridization or NGS [30]. Their results showed that the euploid rates of 1PN blastocysts from IVF and ICSI did not differ significantly. The current study also found that the euploid rates of 1PN and 2PN blastocysts derived from ICSI cycles were not significantly different, which is consistent with the previous studies [33, 34]. Importantly, all the euploid 1PN blastocysts were diploid. This observation further supports the potential use of 1PN blastocysts from ICSI cycles. Our results showed that 1PN blastocysts from IVF and ICSI cycles resulted in a comparable CPR, LBR and neonatal outcome with 2PN blastocysts. No congenital malformations were found in newborns developed from 1PN blastocysts. Although the sample size was limited in the current study, it appeared that 1PN embryos reaching the blastocyst stage from ICSI cycles could achieve similar clinical outcomes to blastocysts from IVF. Therefore, 1PN blastocysts from both IVF and ICSI cycles could be selected for transfer similarly to 2PN blastocysts.

This study has several limitations. This is a single-center study, and multi-center studies are needed to further investigate our findings. Moreover, euploid 0PN and 1PN blastocysts diagnosed by NGS underwent subsequent ASA gene chip analysis to detect ploidy alterations, whereas 2PN blastocysts did not. Furthermore, transfer of 0PN and 1PN embryos is uncommon. Therefore, the total numbers of patients who underwent transfer of such embryos were limited, and the safety of 0PN and 1PN blastocyst transfer requires further exploration.

Overall, our results indicate that 0PN and 1PN blastocyst transfers can result in similar clinical outcomes to 2PN blastocysts. This finding may increase future opportunities of obtaining pregnancy, because it is possible to transfer 0PN and 1PN blastocysts for patients who do not produce or have difficulty providing 2PN embryos. The 0PN and 1PN blastocysts derived from ICSI cycles can be used the same as those from IVF cycles.

## Data Availability

The data sets used and analyzed during the current study are not publicly available but are available from the corresponding author on reasonable request.
